# Monocyte-derived and M1 macrophages from ankylosing spondylitis patients released higher TNF-α and expressed more *IL1B* in response to BzATP than macrophages from healthy subjects

**DOI:** 10.1038/s41598-021-96262-2

**Published:** 2021-09-08

**Authors:** Maryam Akhtari, Seyed Jalal Zargar, Mahdi Vojdanian, Ahmadreza Jamshidi, Mahdi Mahmoudi

**Affiliations:** 1 Department of Cell and Molecular Biology, School of Biology, College of Science, University of Tehran, P.O. Box: 141556455, Tehran, Iran; 2 Rheumatology Research Center, Shariati Hospital, Tehran University of Medical Sciences, Kargar Ave, P.O. Box: 1411713137, Tehran, Iran; 3 Inflammation Research Center, Tehran University of Medical Sciences, Tehran, Iran

**Keywords:** Cell biology, Ankylosing spondylitis

## Abstract

Macrophages participate in the pathogenesis of ankylosing spondylitis (AS) by producing inflammatory cytokines. Extracellular adenosine triphosphate (eATP), released during cell stress, acts through purinergic receptors (P2XR and P2YR) and induces inflammatory responses. We investigated the effect of 2ʹ(3ʹ)-O-(4-benzoyl benzoyl) ATP (BzATP) (a prototypic agonist of P2X7R) on the production of inflammatory cytokines in both monocyte-generated (M2-like) and M1 macrophages from patients and controls. Macrophages were differentiated from isolated periphery-monocytes (n = 14 in each group) by macrophage colony-stimulating factor (M-CSF). Using LPS and IFN-γ, macrophages were skewed toward M1 type and were treated with BzATP. Gene expression and protein release of IL-1β, IL-23, and TNF-α were evaluated by real-time PCR and ELISA methods respectively before and after treatment. BzATP significantly increased the protein release of TNF-α and the expression of *TNFA* and *IL1B* in monocyte-generated macrophages. Besides, BzATP treatment significantly upregulated *IL1B* expression, reduced *TNFA* and *IL23A* expression, and TNF-α release in M1 macrophages from both groups. Monocyte-generated and M1 macrophages from AS patients released higher TNF-α and expressed more *IL1B* in response to the same concentration of BzATP treatment respectively. Based on our results, AS macrophages were more sensitive to BzATP treatment and responded more intensively. Besides, the diverse effects of BzATP on monocyte-derived and M1 macrophages in our study may represent the differed inflammatory properties of these two groups of macrophages in response to eATP in the body.

## Introduction

Under the normal physiological state, adenosine triphosphate (ATP) exists at a low level in the extracellular space, however, under cellular stress or damage, it is released in large amounts to the outside milieu^1,2^. Extracellular nucleotides and their derived nucleosides (such as adenosine) participate in purinergic signaling which is an evolutionarily conserved signaling pathway and is involved in various pathophysiological events including neuromodulation, cell differentiation, migration, immune responses, and inflammation^3^. During inflammation, ATP is released from activated leukocytes by passive leakage or active release through Pannexin 1 channels^4^. Extracellular ATP acts through type 2 purinergic receptors (P2R) including P2X ligand-gated ion channels and G protein-coupled receptors^5^. P2X7 is the most studied P2R in the innate and adaptive immunological pathways and is thought to be responsible for the NLR family pyrin domain-containing (NLRP)-3 inflammasome activation and the maturation and secretion of inflammatory cytokines such as interleukin (IL)-1β and IL-18 of immune cells especially macrophages^6^. In addition, extracellular ATP-dependent P2X7 activation can induce cellular death and apoptosis via caspases activation^7^.

Macrophages as the effector cells of innate immunity have a fundamental role in the induction and resolution of inflammation. Macrophages have been classified into two groups, M1 (classically activated) that produce inflammatory cytokines and participate in autoimmune responses, and M2 (alternatively activated) which participate mainly in tissue remodeling^8,9^. The role of extracellular ATP in the macrophages' activation and function has been demonstrated in the different in vitro and in vivo studies^10–12^. Macrophage treatment with lipopolysaccharides (LPS) and ATP resulted in the NLRP3 inflammasome activation and production of IL-1β and IL-18 in cell culture^13^.

Ankylosing spondylitis (AS) is a type of axial inflammatory arthritis called spondyloarthritis (SpA)^14^. The disease involves mainly the spine and sacroiliac joints and is characterized by long-term inflammation, inflammatory destruction, and post-new bone formation of spinal vertebrae^15^. Environmental and genetic factors including human leukocyte antigene (HLA)-B27 positivity are contributed to the increased risk of disease^16–18^. Studies demonstrated a central role of the tumor necrosis factor (TNF)-α and IL-23/IL17 axis in the inflammation and pathogenesis of AS^19^. Macrophages participate in the AS pathogenesis by producing a large amount of inflammatory cytokines and are one of the most infiltrated cells in the AS patients’ lesions^20,21^. It is also demonstrated that HLA-B27 misfolding induces endoplasmic reticulum (ER) stress and increases IL-23 production in macrophages and immune dysregulation^22^.

During the pathological condition, the level of extracellular ATP is increased and ATP-dependent P2X7R activation induces the inflammatory responses in macrophages. Concerning the importance of macrophages in the inflammatory responses of AS disease, here we investigated the effect of a prototypic agonist of P2X7R (BzATP) on the production of inflammatory cytokine in both monocyte-generated (M2-like) and M1 macrophages from AS patients in contrast to macrophages from healthy subjects.

## Results

### Extracellular ATP in the supernatant of monocyte-generated macrophages

The level of eATP in the supernatant of M2-like macrophages from AS patients and controls was 2.6 ± 1.6 μM and 3.3 ± 2.1 μM respectively (Supplementary Fig. S1) and there was not a notable difference between patients and controls.

### The gene expression and protein secretion of inflammatory cytokines in monocyte-derived and M1 macrophages

First, we measured the level of mRNA expression and protein secretion of IL-23, IL-1β, and TNF-α pro-inflammatory factors in monocyte-generated macrophages and M1 macrophages from AS and healthy individuals. As we reported earlier, M2-like macrophages of patients produced higher IL-23 compared to control macrophages^23^. We did not find remarkable differences in the gene expression and protein production of TNF-α and IL-1β in M2-like macrophages between controls and patients.

**Figure 1:**
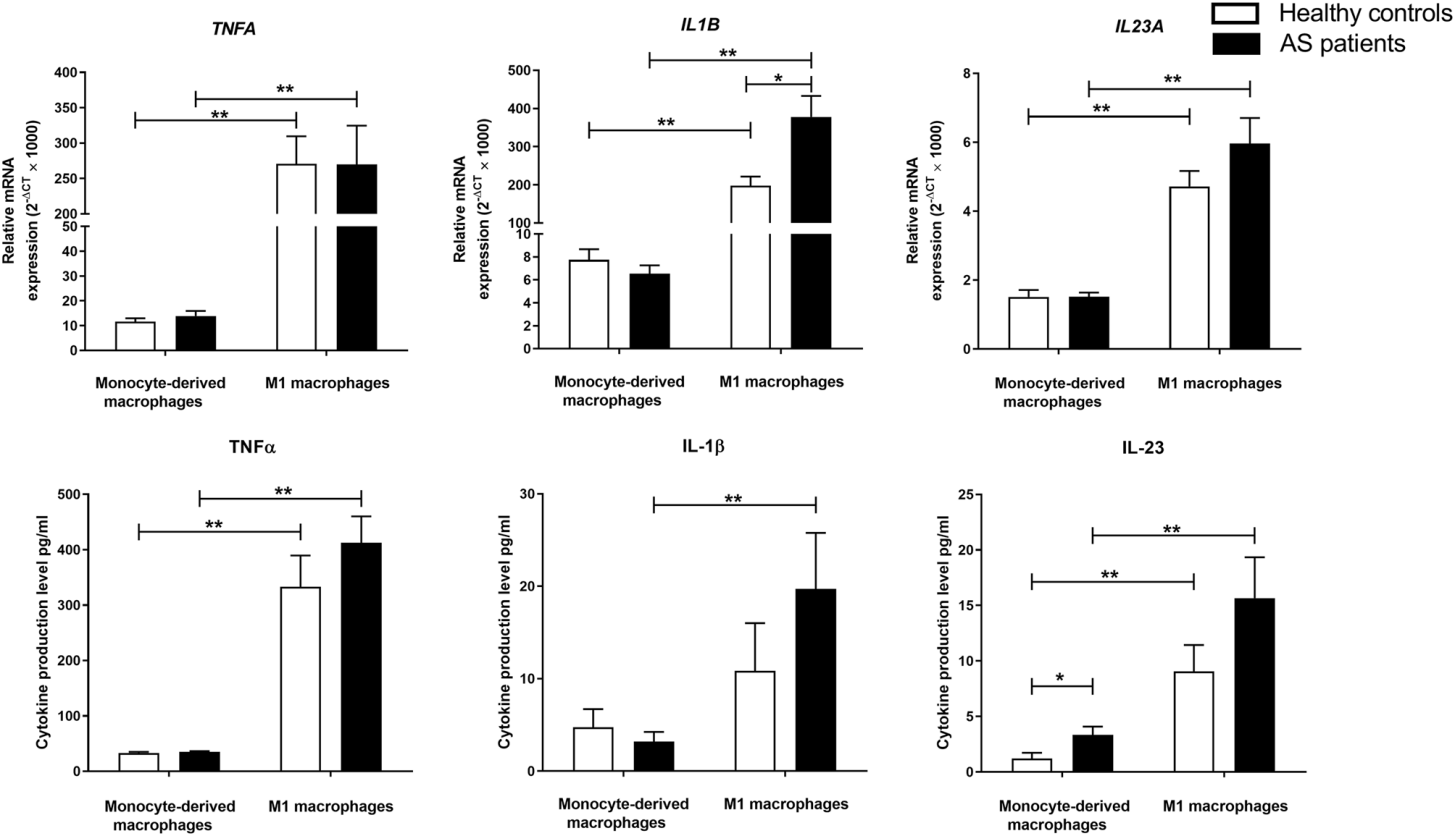
The mRNA expression and the protein production of pro-inflammatory cytokines in monocyte-generated and M1 macrophages from AS and healthy individuals. M1 macrophages from AS patients significantly expressed an elevated level of *IL1B* gene. The gene expression levels of *IL1B*, *TNFA*, and *IL23A* and the protein production levels of IL-23 and TNF-α were significantly elevated in macrophages after their differentiation to M1 type. However, only macrophages from AS patients produced a significantly elevated level of IL-1β after differentiation to M1 type. The data are presented as the mean ± SEM (*P ≤ 0.05, **P ≤ 0.01, ***P ≤ 0.001).

The gene expression of *IL1B*, *TNFA*, and *IL23A* and the protein release of IL-23 and TNF-α were notably elevated in macrophages after their differentiation to M1 type in both AS patients and controls (*P*-value < 0.01 for all). However, only macrophages from AS patients secreted a significantly elevated level of IL-1β after differentiation to M1 type (*P-value* < 0.01). M1 macrophages from AS patients significantly expressed an elevated level of *IL1B* gene (*P-value* < 0.05) compared to controls. Although the secretion level of IL-1β was also upregulated in AS M1 macrophages compared to controls, the difference was not significant. Besides, we did not find notable differences in the expression and production level of IL-23 and TNF-α between M1 macrophages from AS patients and controls. The relative gene expression and protein secretion levels of studied pro-inflammatory cytokines in monocyte-derived and M1 macrophages of patients and controls are shown in Table 1 and Fig. 1.

**Table 1:** The mRNA expression and protein production of IL-23, IL-1β, and TNF-α pro-inflammatory cytokines in monocyte-derived (M2-like) and M1 macrophages from AS patients and healthy controls.

	CO M2-like macrophages (N = 14)	CO M1 macrophages (N = 14)	AS M2-like macrophages (N = 14)	AS M1 macrophages (N = 14)	CO M1/M2-like	AS M2-like/CO M2-like	AS M1/M2-like	AS M1/CO M1
	Relative gene expression				Fold change			
*TNFA*	11.63 ± 4.95	270.67 ± 129.00	13.88 ± 7.72	269.84 ± 204.70	23.27	1.19	19.44	1.00
*IL1B*	7.74 ± 3.35	197.94 ± 85.70	6.53 ± 2.67	377.98 ± 206.43	25.57	0.84	57.91	1.91
*IL23A*	1.51 ± 0.70	4.72 ± 1.68	1.52 ± 0.43	5.97 ± 2.55	3.12	1.00	3.93	1.26

	Protein production (pg/ml)				Fold change			
TNF-α	33.04 ± 6.66	332.95 ± 211.25	34.84 ± 5.47	412.26 ± 172.90	10.08	1.05	11.83	1.24
IL-1β	4.74 ± 6.84	10.86 ± 18.54	3.20 ± 3.60	19.73 ± 22.63	2.29	0.68	6.17	1.82
IL-23	1.22 ± 1.83	9.05 ± 8.91	3.34 ± 2.85	15.66 ± 13.25	7.42	2.74	4.69	1.73

Data are presented as mean ± SD. *AS* ankylosing spondylitis;* CO* controls;* TNF-α* tumor necrosis factor-alpha,* IL-1β* interleukin 1 beta,* IL-23* interleukin-23.

### The gene expression and protein secretion of inflammatory cytokines in monocyte-generated macrophages after BzATP treatment

**Figure 2:**
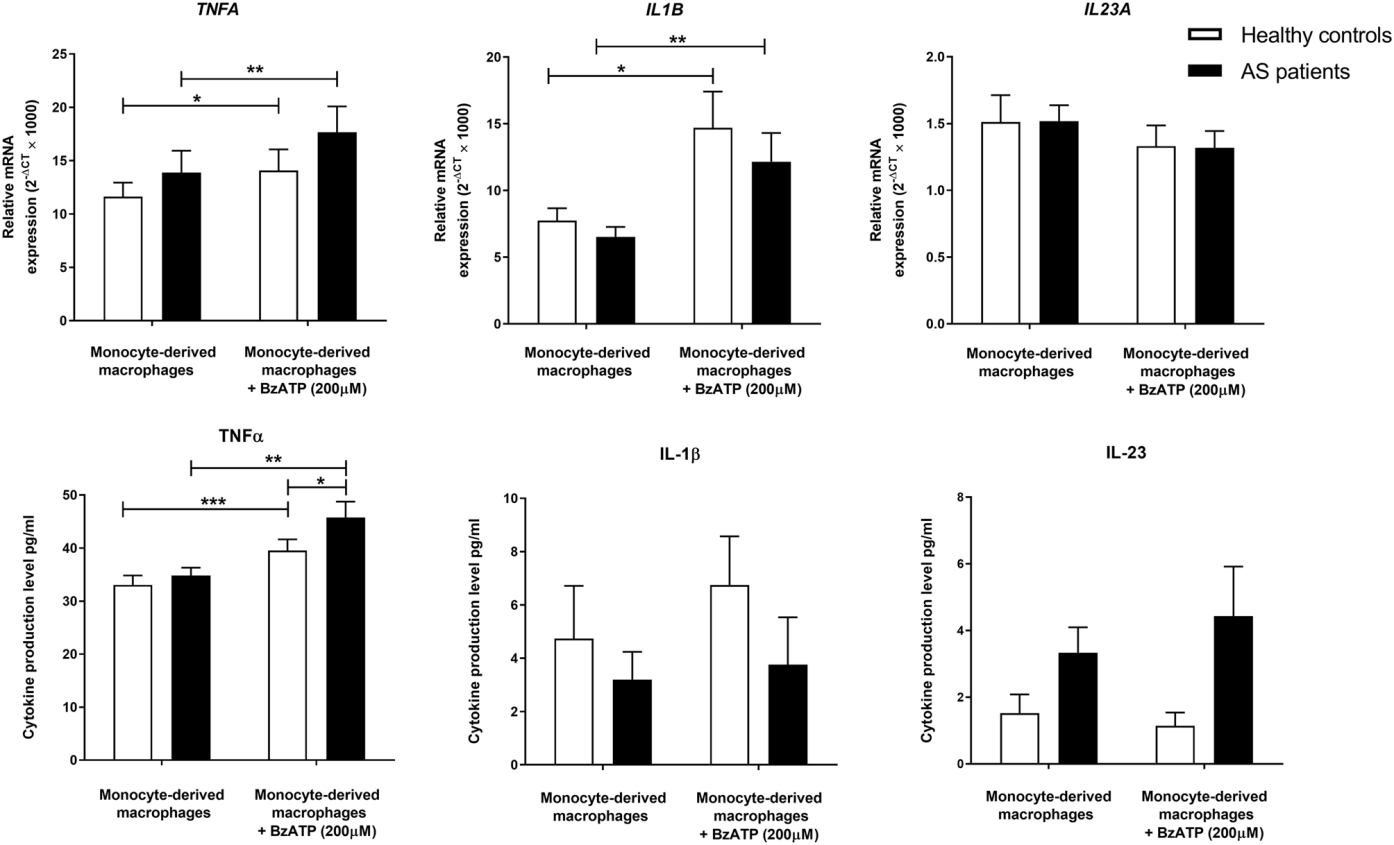
The effect of BzATP treatment on the expression and production of pro-inflammatory cytokines in monocyte-generated macrophages from patients and controls. BzATP significantly increased the mRNA expression level of *TNFA* in AS and healthy macrophages. The production level of TNF-α was also significantly upregulated after BzATP treatment in both groups and monocyte-generated macrophages from AS patients significantly produced a higher amount of TNF-α after treatment compared to control ones. Besides, BzATP significantly elevated the expression level of *IL1B* in monocyte-derived macrophages from both patients and controls. The data are presented as the mean ± SEM (*P ≤ 0.05; **P ≤ 0.01; ***P ≤ 0.001).

The effect of 200 μM BzATP treatment on the secretion of pro-inflammatory cytokines in monocyte-derived macrophages from healthy individuals and patients was examined. BzATP significantly elevated the gene expression level of *TNFA* in AS and healthy macrophages (*P*-value < 0.01 and *P*-value < 0.05 respectively). The secretion level of TNF-α was also notably upregulated after BzATP treatment in both groups (*P*-value < 0.01 and *P*-value < 0.001 respectively), however, monocyte-generated macrophages from AS patients significantly released a higher amount of TNF-α after treatment compared to control ones (*P-value* < 0.05). Although BzATP significantly elevated the expression level of *IL1B* in monocyte-generated macrophages from both patients and controls (*P*-value < 0.05 and *P*-value < 0.01 respectively), the elevated protein secretion of IL-1β after treatment did not reach statistical significance. We also did not find any remarkable differences in the expression and release of IL-23 after BzATP stimulation. The relative gene expression and protein secretion levels of studied pro-inflammatory cytokines in monocyte-generated macrophages before and after BzATP treatment are shown in Table 2 and Fig. 2.

**Table 2:** The mRNA expression and protein production of IL-23, IL-1β, and TNF-α pro-inflammatory cytokines in monocyte-derived (M2-like) macrophages from AS patients and healthy controls before and after BzATP treatment.

	CO M2-like macrophages (N = 14)	CO M2-like macrophages + BzATP (N = 14)	CO BzATP treated/untreated	AS M2-like macrophages (N = 14)	AS M2-like macrophage + BzATP (N = 14)	AS BzATP treated/untreated
	Relative gene expression		Fold change	Relative gene expression		Fold change
*TNFA*	11.63 ± 4.95	14.08 ± 6.57	1.21	13.88 ± 7.72	17.65 ± 8.44	1.27
*IL1B*	7.74 ± 3.35	14.70 ± 9.42	1.90	6.53 ± 2.67	12.16 ± 7.47	1.86
*IL23A*	1.51 ± 0.70	1.33 ± 0.52	0.88	1.52 ± 0.43	1.32 ± 0.37	0.87

	Protein production (pg/ml)		Fold change	Protein production (pg/ml)		Fold change
TNF-α	33.04 ± 6.66	39.53 ± 7.29	1.20	34.84 ± 5.47	45.76 ± 9.97	1.31
IL-1β	4.74 ± 6.84	6.75 ± 6.04	1.42	3.20 ± 3.60	3.76 ± 5.64	1.17
IL-23	1.22 ± 1.83	1.14 ± 1.44	0.93	3.34 ± 2.85	4.43 ± 5.14	1.33

Data are presented as mean ± SD. *AS* ankylosing spondylitis; *CO* controls; *TNF-α* tumor necrosis factor-alpha, *IL-1β* interleukin 1 beta, *IL-23* interleukin-23.

### The gene expression and protein secretion of inflammatory cytokines in M1 macrophages after BzATP treatment

**Figure 3:**
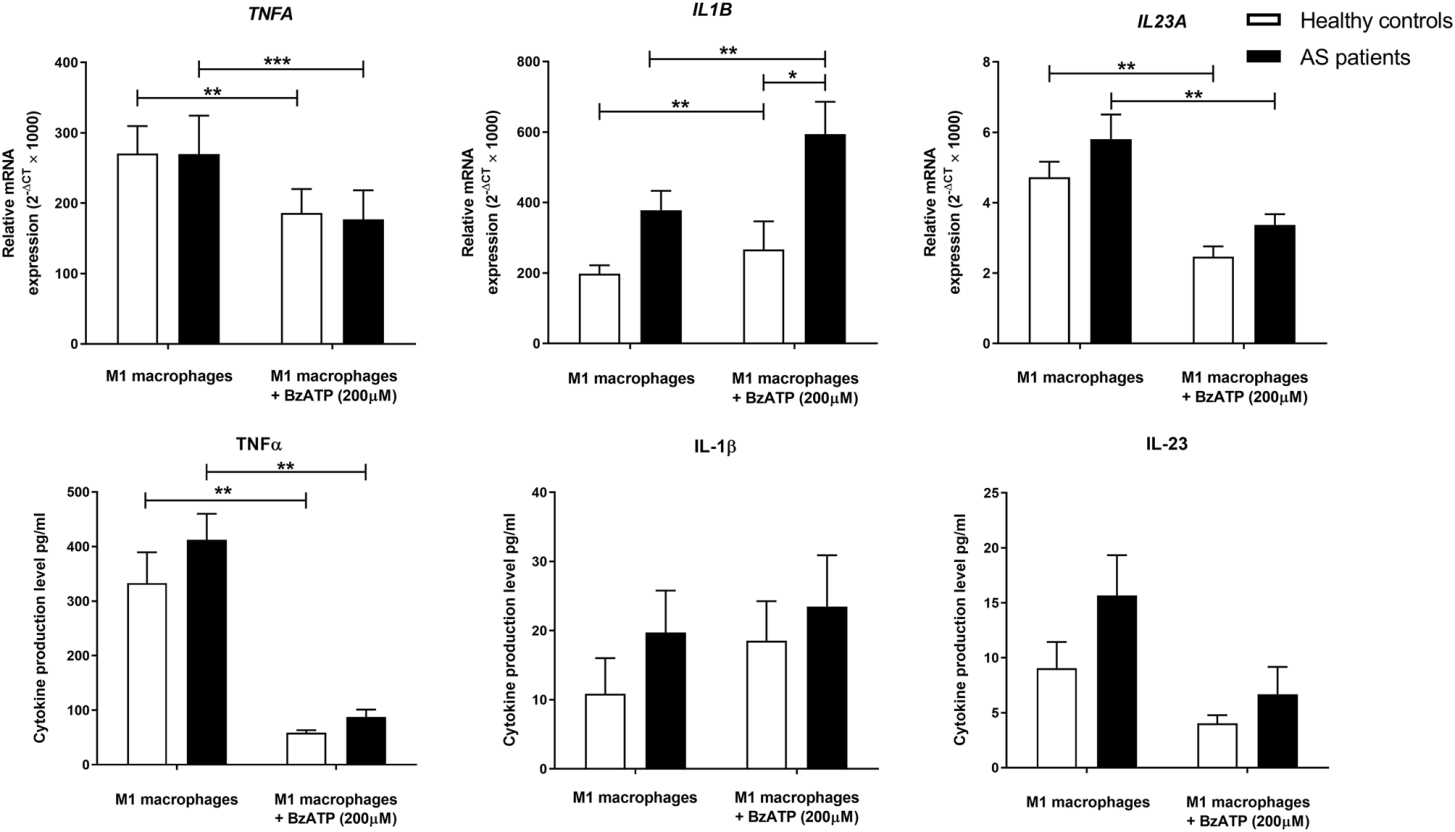
The effect of BzATP treatment on the production of pro-inflammatory cytokines in M1 macrophages from patients and controls. BzATP treatment significantly upregulated *IL1B* gene expression in M1 macrophages from both groups of macrophages. AS M1 macrophages significantly expressed higher *IL1B* mRNA in response to BzATP treatment compared to controls. Interestingly, BzATP significantly diminished the expression and production of TNF-α and the expression of *IL23A* in both healthy and AS M1 macrophages. The data are presented as the mean ± SEM (*P ≤ 0.05, **P ≤ 0.01, ***P ≤ 0.001).

We also assessed the effect of 200 μM BzATP on the secretion of the pro-inflammatory cytokines in AS and healthy M1 macrophages. BzATP treatment significantly upregulated *IL1B* gene expression in M1 macrophages from both groups (*P*-*value* < 0.01 for both). It was also found that AS M1 macrophages significantly expressed higher *IL1B* mRNA in response to BzATP treatment compared to controls (*P*-*value* < 0.05). The effect of BzATP on the secretion of IL-1β in M1 macrophages was not significant. Interestingly, BzATP significantly reduced the expression and secretion of TNF-α in both healthy and AS M1 macrophages (*P*-*value* < 0.01 for all). It was also found that BzATP meaningfully reduced *IL23A* gene expression in M1 macrophages from controls and patients groups (*P*-*value* < 0.01 for both) but the change in IL-23 secretion was not significant. The relative gene expression and protein secretion levels of studied pro-inflammatory cytokines in M1 macrophages before and after BzATP treatment are shown in Table 3 and Fig. 3.

**Table 3:** The mRNA expression and protein production of IL-23, IL-1β, and TNF-α pro-inflammatory cytokines in M1 macrophages from AS patients and healthy controls before and after BzATP treatment.

	CO M1 macrophages (N = 14)	CO M1 macrophages + BzATP (N = 14)	CO BzATP treated/untreated	AS M1 macrophages (N = 14)	AS M1 macrophages + BzATP (N = 14)	AS BzATP treated/untreated
	Relative gene expression		Fold change	Relative gene expression		Fold change
*TNFA*	270.67 ± 129.00	186.08 ± 113.22	0.69	269.84 ± 204.7	176.94 ± 154.63	0.66
*IL1B*	197.94 ± 85.70	267.17 ± 286.55	1.35	377.98 ± 206.43	594.10 ± 343.33	1.57
*IL23A*	4.72 ± 1.68	2.46 ± 1.06	0.52	5.97 ± 2.55	3.37 ± 0.97	0.56

	Protein production (pg/ml)		Fold change	Protein production (pg/ml)		Fold change
TNF-α	332.95 ± 211.25	58.88 ± 15.70	0.18	412.26 ± 172.90	87.41 ± 48.87	0.21
IL-1β	10.86 ± 18.54	18.54 ± 21.43	1.71	19.73 ± 22.63	23.48 ± 27.75	1.19
IL-23	9.05 ± 8.91	4.02 ± 2.74	0.44	15.66 ± 13.25	6.67 ± 8.67	0.43

Data are presented as mean ± SD. *AS* ankylosing spondylitis; *CO* controls; *TNF-α* tumor necrosis factor-alpha, *IL-1β* interleukin 1 beta, *IL-23* Interleukin-23.

### The cellular death rate of BzATP treated monocyte-generated and M1 macrophages

The cellular death rate of monocyte-derived and M1 macrophages treated with 200 µM BzATP was also investigated and is shown in Supplementary Figure S2. The cellular death rate of M2-like macrophages after BzATP treatment was 17.6% and the cellular death rate of M1 macrophages before and after treatment was 12.6% and 32.5% (*P* < 0.05) respectively.

## Discussion

Macrophages play a substantial role in the pathogenesis of rheumatic diseases and are predominant in SpAs inflammatory lesions^21^. They participate in the induction of autoimmunity and autoinflammatory responses of AS^24,25^. While M2 macrophages are involved in tissue remodeling and destruction and resolving the inflammation, M1 macrophages are known for producing a high amount of inflammatory mediators and can induce Th1 immunity^26^. Previous research showed that M1 macrophage polarization with LPS alone or in combination with IFN-γ leads to the release of an elevated amount of inflammatory cytokines including TNF-α, IL-23, IL-12, and IL-1β^27–29^. In line with previous research, in our study, the level of gene expression and protein secretion of TNF-α and IL-23 was significantly upregulated in both AS and healthy macrophages by polarization toward M1 type. However, we found that only M1 macrophages from AS patients and not healthy macrophages produce an increased amount of IL-1β after polarization. We also compared the level of inflammatory cytokines between patients' and controls' macrophages. Although M1 macrophages from AS patients expressed and produced higher levels of IL-23, IL-1β, and TNF-α compared to healthy M1 macrophages, the increase did not reach statistical significance except for *IL1B* expression. We also did not find a significant difference in TNF-α production between AS and healthy macrophages. Nevertheless, different studies reported increased production levels of TNF-α from peripheral blood mononuclear cells (PBMCs) and M1 macrophages of AS patients^25,30^. Regarding our results, it seems that IL-1β expression and secretion are dysregulated in M1 macrophages of AS patients. In line with our results, Gu et al. found that *IL1B* was highly expressed in PBMCs from SpA patients compared to healthy ones^31^. Besides in a meta-analysis study of microarray datasets from AS/SpA patients, it was demonstrated that the expression of *IL1B* was elevated in the blood samples of patients^32^. According to the increased IL-23 production in monocyte-derived macrophages and elevated *IL1B* expression in M1 macrophages of AS patients, it seems that macrophages from AS patients display an upregulated inflammatory phenotype compare to healthy ones.

It has been demonstrated that extracellular ATP acts as a danger signaling molecule through activation of purinergic receptors on the cell surface and can trigger inflammatory cascades in macrophages^10,12^. Therefore, we compared the level of extracellular ATP between AS macrophages and healthy ones. We did not find a remarkable difference. The concentration of extracellular ATP in the macrophages supernatant was approximately 3 μM in our study which is higher than the concentration reported in previous studies which are about 10 nM under physiological conditions^33^. To the best of our knowledge, it is the first study that assessed the concentration of eATP in primary macrophages environment and more investigations are needed.

Previous reports have suggested a critical role for P2X7 purinergic receptor on the activation, polarization, and inflammatory cytokines' production of macrophages^34^. P2X7R activation induces ATP release to the cell-extracellular environment through activation of pannexin 1 (PANX1) hemichannels in a positive feedback loop and amplifies inflammatory signaling pathways^35^. Previously we reported that the expression of *PANX1* and *P2RX7* did not significantly differ between M2-like macrophages from patients and controls^36^. So Due to the role of macrophages and their inflammatory cytokines in AS pathogenesis, we aimed to consider the effects of BzATP as a prototypic P2X7R agonist on the expression and release of inflammatory cytokines in monocyte-derived (M2-like) and LPS and IFN-γ-primed M1 macrophages from AS patients.

It has been suggested that LPS from gram-negative bacteria induces the biosynthesis of pro-IL-1β in macrophages and eATP via P2X7R induces the NLRP3 inflammasome assembly, activates caspase-1, and releases IL-1β^37,38^. In our study, BzATP induced the expression of *IL1B* in both monocyte-derived and M1 macrophages of AS and control individuals. Although the treatment induced IL-1β release in M2-like and M1 macrophages, an increase was not significant in neither groups. This may be due to our limited sample size and the small amount of released IL-1β. Besides, in the current study, BzATP showed bi-functional effects on TNF-α expression and release from macrophages. It increased the gene expression and protein release of TNF-α in monocyte-generated macrophages of both AS and healthy groups. However, the treatment diminished TNF-α release in LPS and IFN-γ-primed M1 macrophages of AS patients and controls. Our results are different from the previous studies showing that P2X7R activation by eATP induces TNF-α release by LPS-primed macrophages^39,40^. In line with our study, Sala et al. realized that eATP in a dose-dependently manner inhibits LPS-induced TNF-α production of dendritic cells (DCs)^41^. Kucher et al. also demonstrated that BzATP (100 or 1000 µM) or a high dose of eATP attenuates TNF-α release from rat LPS-primed cortical astrocytes^42^. Although they did not see any effect of BzATP and LPS treatment on cell viability of astrocytes, in our study BzATP in combination with LPS and IFN-γ significantly reduced cell viability of primary macrophages.

The effect of eATP on the production of IL-23 and IL-12 family members has not been deeply studied. It is suggested that eATP inhibits IL-12 production^41^ and IL-27 expression by DCs^43^. Schnurr et al. found that eATP enhances IL-23 expression in DCs or LPS-primed DCs^43^. Besides, Paustian et al. found that eATP and activation of toll-like receptor 2 (TLR2) induce the release of IL-23 from cultured monocytes^44^. In a study by Diaz-Perez et al., intradermal injection of BzATP into mice did not induce notable inflammatory responses, though BzATP in combination with POM1, an inhibitor of ecto-nucleoside triphosphate diphosphohydrolases (E-NTPDase), resulted in a significant increase in IL-23 production^45^. In our study, BzATP treatment did not influence the expression and secretion of IL-23 in monocyte-generated macrophages. Besides, LPS-IFN-γ primed M1 macrophages from controls and AS patients expressed reduced *IL23A* gene in response to BzATP treatment*.* Although most of the studies reportd the effect of BzATP on LPS-primed macrophage at protein release level, here we showed that BzATP alone or in combination with LPS and IFN-γ can affect TNF-α, IL-1β and IL-23 at gene expression level on macrophages.

Previous research found that eATP in combination with LPS may induce cell death in macrophages by activating caspase 1^44,46^. Extracellular ATP has been shown to have growth inhibitory and systemic cytotoxic activity on different cell types^47–49^. The effect of eATP depends on the cell types, its concentration, and the time of exposure^50^. Due to its cytotoxicity, it was considered as an alternative therapeutic option for the treatment of malignancies^51^. In our study, it was shown that BzATP treatment, along with LPS and IFN-γ significantly increases the death rate in macrophages. While the expression of *IL1B* was increased in this group, IL-23 and TNFα expression and production level were diminished, this may be due to the increased death rate of treated cells. It seems that the use of eATP can induce cell death and diminish inflammatory cytokine production in classically activated macrophages, however considering the systemic cytotoxic effects of eATP, discovering new specific analogs with a less cytotoxic activity which could be applied in future systemic treatments.

Results from the current study showed that monocyte-derived and M1 macrophages of AS patients released higher TNF-α and expressed more *IL1B* in response to the same concentration of BzATP treatment respectively. It seems that macrophages from AS patients display elevated inflammatory reactions in response to BzATP. In the literature, there are no previous studies regarding P2X7R activation and extracellular ATP in AS patients and there is only a recent publication on P2X7R genetic susceptibility in AS. In a study by Pan et al., it is found that rs7958311 A allele in the *P2RX7* gene shows a protective effect on AS incidence in females and is associated with higher disease activity in male patients^52^. However, some studies evaluated the effect of eATP in other rheumatic diseases. Li et al. reported that BzATP stimulation of CD4^+^ PBMCs from rheumatoid arthritis (RA) and systemic lupus erythematosus (SLE) patients results in higher Ca2 + influx compared to controls^53^. Castrichini et al. also found that LPS-primed monocytes from patients with Behcet disease (BD), release elevated IL-1β in response to BzATP than controls^54^. Monaco et al. investigated the effect of eATP on the fibroblast of patients with systemic sclerosis (SSc). They found that patient’s fibroblasts are at a pre-activated state and eATP treatment induces IL-6 release even in the absence of LPS priming. Moreover, both Ca2^+^ influx and release occurred at lower ATP concentrations in SSc fibroblasts^55^. Gentile et al. also reported higher Ca^2+^ permeability in response to eATP in SSc fibroblasts than in controls^56^. Based on the results, it seems that fibroblasts and immune cells from various rheumatic diseases are more sensitive to the presence of eATP and inflammatory environments compared to healthy ones. Therefore, controlling the level of eATP and targeting its signaling pathway can be a new therapeutic option for treating these patients.

## Conclusion

In conclusion, diverse observed effects of BzATP on monocyte-derived and M1 classically activated macrophages in our study, especially regarding TNF-α and IL-23, may represent the differed inflammatory properties of these two groups of macrophages in response to environmental ATP in the body. Besides, it seems that the cytotoxicity activity of BzATP on classically activated macrophages may affect their production of inflammatory cytokines. In addition, according to our results, AS macrophages were more sensitive to BzATP treatment, responded more intensively and expressed an increased level of *IL1B,* and released elevated TNF-α in response to the same concentration of treatment. Therefore, it is expected that alternation in this signaling pathway may contribute to the pathogenesis of AS disease, and targeting this pathway can consider as a therapeutic option in the future. There are no other reports about the effects of eATP and activation of purinergic signaling on macrophages from AS patients. Due to the obtained results in this research, more research with a bigger sample size on different types of immune cells is needed for discovering the exact pathogenic role of this signaling pathway in AS disease.

## Methods and patients

### Study participants

In this research, 30 ml of venous blood were drawn from AS patients and sex/age-matched healthy volunteers (11 males and 3 females). Patients were recruited from the outpatient rheumatology clinic, Shariati Hospital, Tehran, Iran. Patients were included based on the modified New York classification criteria^57^ and had Bath Ankylosing Spondylitis Disease Activity Index (BASDAI) more than four. Due to the significant influence of different biological therapy on the production of inflammatory cytokines, treatment-naive patients were chosen for this study. Besides, healthy volunteer participants did not have a previous familial or personal history of rheumatic disorders or any other autoimmune diseases. The clinical and demographical details of patients and healthy contributors are summarized in Table 4. This research was conducted based on the Declaration of Helsinki guidelines and was approved by the Ethics Committee of Tehran University of Medical Sciences (Approval No: IR.TUMS.REC.1394.1504). All participants provided signed written informed consent before their contribution to the study.

**Table 4:** Clinical and demographical features of healthy individuals and AS patients.

Group	CO individuals (n = 14)	AS patients (n = 14)
Female/male (%)	3/11 (21/79%)	3/11 (21/79%)
Age, years	32 ± 9.6	32 ± 10
Smoking, %	35	35
ESR, mm/h (SD)	5 (3)	37 (19)
Disease period, years	–	7.5 ± 6
HLA-B27 positivity, %	–	71
BASMI score (SD)	–	3.7 (2.5)
BASDAI score (SD)	–	6.1 (1.9)
BASFI score (SD)	–	4.6 (2.7)
PDGA score‌ (SD)	–	7 (2.7)
BASG score (SD)	–	7.2 (1.8)
ASQoL score (SD)	–	9.5 (5.6)
Biological agents, %	0	0
Methotrexate, %	0	0

*AS* ankylosing spondylitis, *CO* control, *ESR* erythrocyte sedimentation rate, *HLA-B27* human leukocyte antigen (subtypes B*2701-2759), *BASMI* Bath Ankylosing Spondylitis Metrology Index, BASFI: Bath Ankylosing Spondylitis Functional Index, BASDAI: Bath Ankylosing Spondylitis Disease Activity Index, BAS-G: Bath Ankylosing Spondylitis Global Score, *PDGA* Patient’s disease global assessment, *ASQoL* Ankylosing spondylitis quality of life, *SD* standard deviation.

### Monocyte separation, macrophage differentiation, and culture

Ethylenediaminetetraacetic acid (EDTA) containing tubes were used to collect venous blood. Collected samples were processed in less than 4 h. The PBMCs were separated from 1:2 diluted blood with phosphate-buffered saline (PBS; GIBCO Invitrogen) using Ficoll density gradient centrifugation. To separate the monocytes, extracted PBMCs were washed by PBS solution and incubated with CD14 microbeads. Positive selection for CD14 marker undertook using magnetic-activated cell sorting (MACS) methods (Miltenyi Biotec). The purity of isolated monocytes was analyzed by flow cytometry using anti-CD14 antibodies (phycoerythrin (PE)-conjugated; BD bioscience) and 92–95% of isolated cells were CD14 positive^58^. To differentiate monocytes toward macrophages, the separated CD14^+^ monocytes were seeded on 24-well plates at a concentration of 5 × 10 ^5 cells per well in Roswell Park Memorial Institute (RPMI) medium supplemented with recombinant macrophage-colony stimulating factor (50 ng/ml M-CSF; eBioscience) for 1 week. The culture media also included L-glutamine (2 mM, Biosera), penicillin (100 U/ml, Sigma), streptomycin (100 μg/ml, Sigma), and 10% fetal bovine serum (FBS; Gibco BRL). CD206 and CD163 macrophages' surface markers were assessed by flow cytometry with appropriate antibodies and isotype controls and the purity was 95 and 97% respectively^58^.

### M1 macrophage polarization

Using LPS (100 ng/ml; Sigma) and interferon-gamma (1000 units/ml IFN-γ; R&D systems, MN) for 24 h, monocyte-generated (M2-like) macrophages were polarized toward M1 type. Polarization was confirmed by analysis of the transcriptional phenotype of M2 and M1 macrophages by SYBR green real-time polymerase chain reaction (PCR). After 24 h of incubation with LPS and IFN-γ, the gene expression of M1 macrophage-certain markers (*CXCL11*, *CCR7*, and *IDO*) and M2 macrophage-specific ones (*MRC1* and *CD36*) was notably increased and decreased respectively (Supplementary Fig. S3).

### Extracellular ATP evaluation

Following 7 days of monocyte-incubation with M-CSF, monocyte-generated macrophages from both patients and healthy individuals were seeded on 24-well plates for 24 h. The concentration of secreted ATP in the cell-free supernatant was measured by colorimetric ATP assay kit (Abcam, ab83355) according to the manufacturer’s structure and the absorbance was read at 450 nm with a microplate reader.

### BzATP stimulation and measurement of the expression level of the inflammatory cytokines

M1 and monocyte-generated macrophages from AS patients and healthy individuals were treated with 200 µM of 2ʹ(3ʹ)-O-(4-benzoyl benzoyl) adenosine-5ʹ-triphosphate (BzATP, Sigma-Aldrich), a potent P2X7R agonist. Samples were incubated with or without BzATP for 24 h and compared. Using the High Pure RNA Isolation Kit (Roche), the total RNA was extracted from BzATP treated and non-treated monocyte-derived and M1 macrophages. The complementary DNA (cDNA) was synthesized from an equal amount of total RNA by CellAmp direct RNA prep kit (Takara bio) for real-time PCR. The relative gene expression level of TNF-α (*TNFA*), IL-23 subunit alpha (*IL23A, P19*), IL-1β (*IL1B*), and glyceraldehyde-3-phosphate dehydrogenase (*GAPDH*, as an internal control gene) was measured by SYBR green master mix (Ampliqon) using StepOnePlus™ real-time PCR system (Applied Biosystems). The experiment was done with specific primer sequences for *IL1B* (Forward: 5′ATGGCTTATTACAGTGGCAATGAG3′, Reverse: 5′GTAGTGGTGGTCGGAGATTCG3′), *TNFA* (Forward: 5′CCTGCCCCAATCCCTTTATT3′, Reverse: 5′CCCTAAGCCCCCAATTCTCT3′), *IL23A* (Forward: 5′GGGACAACAGTCAGTTCTGCTT3′, Reverse: 5′TGGGACTGAGGCTTGGAATC3′), and *GAPDH* (Forward: 5′GAGTCAACGGATTTGGTCGT3′, Reverse: 5′GACAAGCTTCCCGTTCTCAG3′). The comparative CT method (2^−ΔΔCT^) was utilized to compare the expression level of cytokines' genes between the BzATP-incubated and control groups.

### Evaluation of cytokine secretion level in macrophages

The secretion level of TNF-α, IL-1β, and IL-23 inflammatory cytokines was investigated by the ELISA method in the supernatant of M2-like and M1 macrophages from healthy individuals and patients before and after BzATP treatment according to manufacturer's protocol (Mabtech).

### Macrophages cell death assessment

To evaluate the effect of BzATP on the cellular death rate of macrophages, the colorimetric MTT assay was performed. The viability of the 200 µM BzATP treated and non-treated monocyte-derived and M1 macrophages was measured after 24 h.

### Statistical analysis

The normality of all data was evaluated by the Kolmogorov–Smirnov normality test. To compare the relative mRNA expression and cytokines secretion between parallel-group, the independent t-test, and paired samples T-test were used for normal distributions and the Mann Whitney test and Wilcoxon tests were used for non-normal distributions. *P-values* less than 0.05 were considered statistically significant. All of the statistical analyses were performed by SPSS version 22 and graphs were prepared by GraphPad Prism 6.

### Ethics approval

This study was performed based on the Declaration of Helsinki guidelines and was approved by the Ethics Committee of Tehran University of Medical Sciences (Approval No: IR.TUMS.REC.1394.1504).

### Consent to participate

The written informed consent was signed by all participants before enrolling in the study.

## Supplementary Information

Supplementary Information.

## Data Availability

All data generated or analyzed during this study are available upon request.
