# Supplementary material for: Monocyte-derived and M1 macrophages from ankylosing spondylitis patients released higher TNF-α and expressed more IL1B in response to BzATP than macrophages from healthy subjects
Source: Sci Rep. 2021 Sep 8;11:17842. doi: 10.1038/s41598-021-96262-2 (PMC8426480; doi:10.1038/s41598-021-96262-2)
Supplement: Supplementary file 1 — Supplementary Information. [file 41598_2021_96262_MOESM1_ESM.pdf]

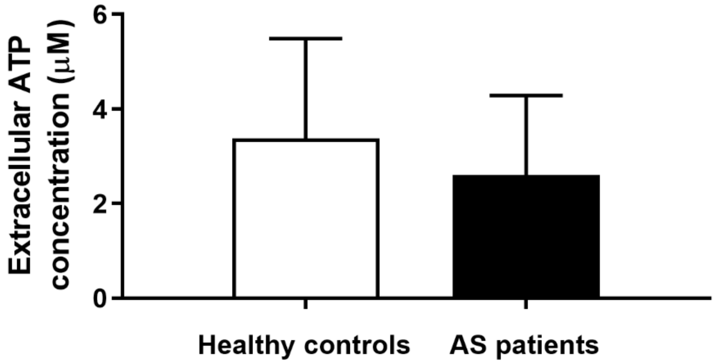

Supplementary Figure S1. The level of extracellular ATP in the supernatant of monocyte-derived macrophages from AS patients and controls. M2-like macrophages from AS patients and healthy persons release 2.6 ± 1.6 μM and 3.3 ± 2.1 μM extracellular ATP into the supernatant respectively. The data is presented as the mean ± SD.

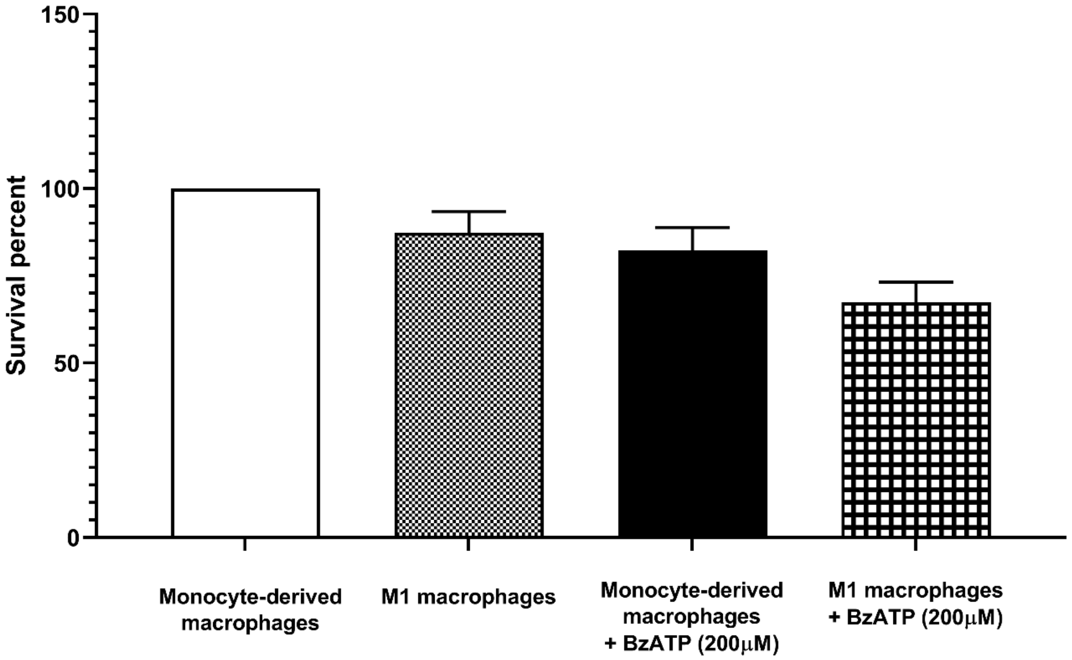

Supplementary Figure S2. The cellular viability of BzATP-treated and untreated monocyte-generated and M1 macrophages assessed by MTT assay. The cellular death rate of monocyte-derived macrophages after 200 μM BzATP treatment was 17.7% and the cellular death rate of M1 macrophages with and without 200 μM BzATP was 32.5% and 12.6% respectively. The data is presented as the mean ± SEM.

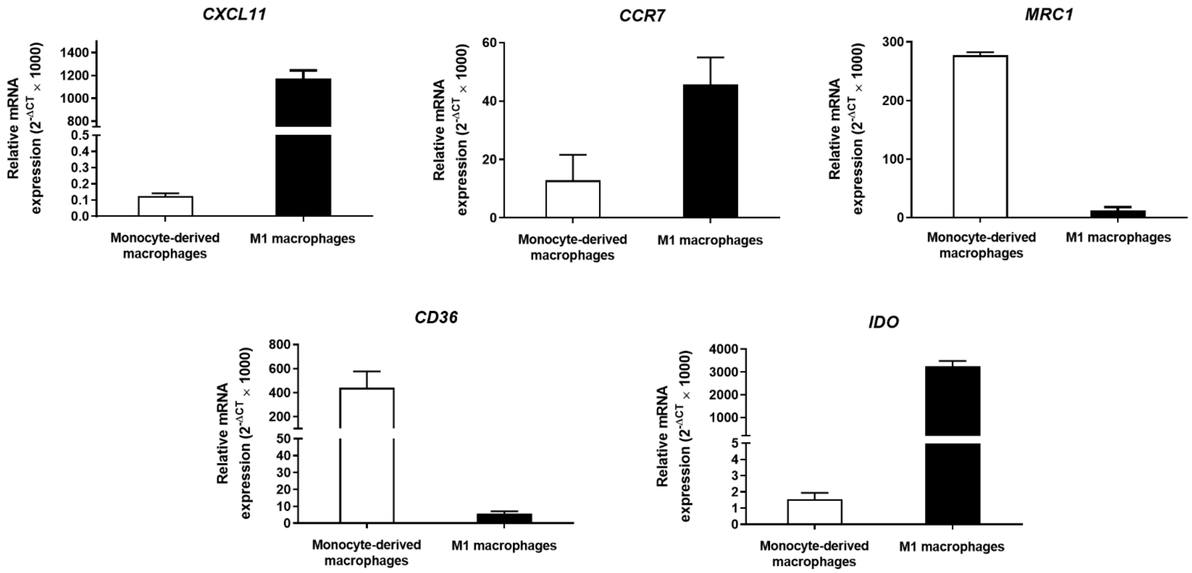

Supplementary Figure S3. Expression of macrophages specific markers in monocyte derived macrophages and M1 macrophages. After 24 hours' incubation with LPS and IFN-γ, the expression of CXCL11, CCR7 and IDO (M1 macrophages specific markers) was increased and the expression of CD36 and MRC1 (M2 macrophage specific markers) was decreased in macrophages.
